# Comparing the frequency, antifungal susceptibility, and enzymatic profiles of the oral fungal composition in patients with and without Alzheimer's disease admitted to a neurology clinic

**DOI:** 10.3389/fcimb.2024.1477230

**Published:** 2024-10-18

**Authors:** Mandana Golipoor, Zahra Rafat, Alia Saberi, Davoud Roostaei, Amir-Mohammad Shabanpour

**Affiliations:** ^1^ Cellular and Molecular Research Center, School of Medicine, Guilan University of Medical Sciences, Rasht, Iran; ^2^ Department of Medical Parasitology and Mycology, School of Medicine, Guilan University of Medical Sciences, Rasht, Iran; ^3^ Neuroscience Research Center, School of Medicine, Guilan University of Medical Sciences, Rasht, Iran; ^4^ Department of Pharmacology, School of Medicine, Guilan University of Medical Sciences, Rasht, Iran; ^5^ Student research committee, Anzali International Medical Campus, Guilan University of Medical Sciences, Guilan, Iran

**Keywords:** oral fungal composition, Alzheimer's disease, mouth, hemolysin, esterase, proteinase

## Abstract

**Background:**

Studies have shown that changes in the frequency of oral microorganisms may play a key role in the development of Alzheimer's disease (AD). However, no research has been conducted on the oral fungal composition in AD-patients. The present study aimed to investigate the changes in the frequency of oral fungal composition, the antifungal susceptibility, and the enzymatic profiles of oral fungal composition in patients suffering from AD compared to non-AD individuals.

**Materials and methods:**

In the present analytical cross-sectional study during 12 months, 76 hospitalized patients with AD were matched with 76 individuals without AD. A sterile serum physiology-moistened cotton-tipped swab was used to sample the mouth area. All swabs were cultured on Sabouraud Chloramphenicol Agar. Fungal identified were confirmed through the PCR-sequencing techniques. Enzyme activity index (EAI) for important pathogenic factors including proteinase, esterase and hemolysin was measured using relevant protocols. The susceptibility to 8 antifungal agents (nystatin, voriconazole, itraconazole, fluconazole, posaconazole, amphotericin B, 5-fluorocytosine, and caspofungin) against fungal strains obtained from AD-patients was evaluated according to the Clinical and Laboratory Standards Institute (CLSI) guidelines, document M38-A2 for filamentous fungi, and document M27-A4 for yeasts.

**Results:**

The results showed that compared to the non-AD individuals, the prevalence of oral fungal composition in AD group was 1.6 times higher. *Candida albicans* was the most common fungal species isolated from oral swab samples of AD group (n=53, 80%) and non-AD group (n=28, 40%), and the diversity of the oral fungal composition in AD-patients were lower than non-AD individuals. Among the 3 investigated virulence factors, a statistically significant difference was shown in terms of hemolysin activity level between the two studied groups (*p*<0.05) and the activity level of esterase and proteinase enzymes did not show a significant difference in the two studied groups (*p*>0.05). The results showed that almost all of the tested isolates were susceptible to nystatin, the most widely prescribed antifungal to treat superficial infections, and only 1.69 % (2/118) of the *Candida* isolates were resistant to this antifungal drug.

**Conclusion:**

Understanding the changes in the frequency of oral fungal composition the antifungal susceptibility, and the enzymatic profiles of oral fungal composition in patients suffering from AD compared to non-AD individuals makes it possible to better understand the etiology of this disease.

## Introduction

Currently, 50 million people in the world are suffering from Alzheimer's disease (AD), and according to global statistics, more than 700 thousand people are suffering from this disease in Iran, and a large part of Alzheimer's disease is misdiagnosed in Iran. In addition, by 2050, the population of people with this disease is expected to reach 113 million (Ashrafizadeh et al., 2021). AD is the most frequent type of dementia, with various signs of progressive decline in memory and cognitive abilities. The characteristic symptom of this disease is the buildup of plaques called beta-amyloid (Aβ) in the brain (Tahami Monfared et al., 2022). This disease causes memory impairment, confusion, mood and behavior changes, and even difficulty walking, speaking, and swallowing. AD was the sixth-leading cause of death among people aged 65 and older in the United States. In patients living with AD, chronic brain inflammation and dysfunction of the immune system are considered the main causes of the disease, which occur several years before the onset of cognitive dysfunction. An effective factor in causing chronic inflammation is pathological alterations in the oral microbiome (Ala et al., 2004; Hoffmann et al., 2009; Cervellati et al., 2020; Onyango et al., 2021; Xie et al., 2022).

The oral microbiome is defined as a community of bacteria (bacterium), viruses (virome), and fungi (mycobiome) that form an ecosystem in the human mouth that maintains health in a state of equilibrium (Radaic and Kapila, 2021). Our knowledge of the human microbiome is still in its infancy, but it has reached a degree of development that shows its influence in the regulation of the immune system and metabolism, allergies, and the treatment and control of serious diseases (Gomaa, 2020; Radaic and Kapila, 2021). The human oral cavity is sterile before birth. During the birth process and rapidly thereafter, microbes from the mother and surrounding environment colonize the oral cavity of the infant. Within weeks, as babies pick up due to more microbes from their family members and surroundings, the types of microbes living in different parts of the body start to specialize and for this reason, oral microbiota composition differs widely between different areas and between different ethnic groups within the same area (Gomaa, 2020; Radaic and Kapila, 2021; Ramezanalipour et al., 2024). The oral mycobiome includes normal fungal flora (*Candida* spp., *Geotrichum* spp., *Trichosporon* spp., and *Rhodotorula* spp.) and non-normal flora fungi (*Mucor* spp., *Rhizopus* spp., *Aspergillus* spp., *Alternaria* spp., *Cladosporium* spp., *Cryptococcus neoformans*, *Fusarium* spp., and other saprophytic fungi). Changes in the composition and frequency of oral microorganisms, especially anaerobic bacteria such as *Treponema*, *Porphyromonas gingivalis*, *Fusobacterium* spp., *Prevotella* spp., and *Actinomyces*, as well as facultative anaerobic *Candida* species, may play a key role in the development of AD (Nasr et al., 2023). Oral yeasts can be found in periodontal pockets, on the mucosae, in root canals, and underneath dentures (denture stomatitis) (Le Bars et al., 2021). Denture stomatitis is common in elderly denture wearers and can be a source of inflammation and systemic infections. It has also been observed that antifungal treatments have reduced the clinical symptoms of some AD patients (Ala et al., 2004; Hoffmann et al., 2009). On the other hand, the resident mycobiome in the mouth needs various virulence factors such as hemolysin, secretory aspartic proteases (Saps), and esterase enzymes, to penetrate deep tissues and cause pathogenesis (Mba and Nweze, 2020; Ramezanalipour et al., 2024). It is noteworthy that each of the fungal species forming the oral mycobiome can have significant differences from others in terms of pathogenicity and antifungal susceptibility (Černáková et al., 2022; Ramezanalipour et al., 2024). However, to date, no research has been conducted on investigating the changes in the composition and frequency of oral mycobiome in patients suffering from AD compared to non-AD individuals. Thus, the present study aimed to investigate the changes in the frequency of oral fungal composition, the antifungal susceptibility, and the enzymatic profiles of oral fungal composition in patients suffering from AD compared to non-AD individuals. Determining the pathogenesis factors of the oral fungal composition in patients suffering from AD compared to non-AD individuals can be an important tool in understanding the pathogenesis of AD.

## Materials and methods

### Ethics statement

Before sampling, informed consent was obtained from the patient or his/her guardian. This study was approved by the ethical committee of Guilan University of Medical Sciences (the number of Ethics Committee protocol: IR.GUMS.REC.1402.194). Furthermore, we reported the basis of our manuscript in line with the STROCSS criteria (Agha et al., 2019).

### Data collection and sampling

This was an analytical cross-sectional study involving patients suffering from AD admitted to a neurology clinic. During a period of 12 months, from June 2023 to June 2024, a total of 76 hospitalized patients with AD were matched with 76 individuals without AD. Within each pair, participants were matched for age (± 5 years) and gender. Information regarding age, gender, body mass index (BMI), AD severity stages (according to the Global Deterioration Scale (GDS), also known as Reisberg Scale) (Reisberg, 1994)), history of receiving broad-spectrum antibiotics, and history of corticosteroid therapy related to each participant was extracted from health records. Patients aged 60 years or older with a diagnosis of probable AD according to the revised NINCDS-ADRDA criteria (Dubois et al., 2007), living with natural teeth and without dentures, and participants who had optimal oral and dental hygiene were included in the present study. Also, elderly patients wearing dentures, patients whose family members did not consent to participation in the research because of mental or behavioral disorders, patients with memory loss that developed suddenly (not gradually), patients with a positive real-time PCR test for COVID-19, patients with severe depression, patients who had received systemic antifungal drugs within the last month, and patients who were diagnosed with oral infections after an intra-oral examination were excluded.

A sterile serum physiology-moistened cotton-tipped swab was used to sample the mouth area (oral mucosa, teeth, gums, and tongue). All swabs were promptly taken to the laboratory in sterile tubes and the microbiological analysis performed immediately. First, the swabs were cultured on Sabouraud Chloramphenicol Agar (SC, Merck, Germany), and then placed in an incubator at 30°C for 3 weeks. The choice of swabs to collect samples was based on their ease of use, non-invasiveness, and ability to precisely obtain samples from specific areas in the oral cavity. The fungal cultures were further characterized by examining their colony morphology, the rate of growth, and preparing Lactophenol cotton blue (LCB) mounts. Yeast isolates were identified based on the production of chlamydoconidia in cornmeal agar (Becton, France) and colony color on chromogenic CHROMagar *Candida* medium (CHROMagar, Paris, France) (Halvaee et al., 2021). Furthermore, for confirmation of identification, all isolates were subjected to PCR and sequencing techniques.

### Molecular technique

#### DNA extraction

Genomic DNA from fungal colonies was extracted using the glass bead disruption method (Behera and Srigyan, 2021).

#### PCR conditions and sequencing

The PCR amplification for each isolate was carried out in the manner that was previously explained (Behera and Srigyan, 2021). The Beta tubulin gene of *Aspergillus* species was amplified using the forward (Bt2a: 5'-GGTAACCAAATCGGTGCTGCTTTC-3') and reverse (Bt2b: 5'- ACCCTCAGTGTAGTGACCCTTGGC-3') primers to differentiate *Aspergillus* isolates at the species level. Also, the universal primers utilized for amplifying fungi at the species level included ITS1 (5′TCC GTA GGT GAA CCT GCG G 3′), which binds to the end of 18S rDNA, and ITS4 (5′TCC TCC GCT TAT TGA TAT GC 3), which binds to the beginning of 28SrDNA. These primers were obtained from Life Technologies in Barcelona, Spain. The Bioneer Advanced Nucleic Acids core facility received the positive PCR products for sequencing. The next step involved using individual sequences for conducting nucleotide–nucleotide searches through the BLASTn algorithm on the NCBI website (https://blast.ncbi.nlm.nih.gov/Blast.cgi). The method used for fungal identifications relied on achieving maximum identities ≥ 99% and query coverage ≥ 98%.

### Antifungal susceptibility testing

Antifungal susceptibility testing was conducted in *in vitro* conditions for strains obtained from AD patients, following the Clinical and Laboratory Standards Institute (CLSI) guidelines. For filamentous fungi, the protocols described in document M38-A2 were used (Clinical and Laboratory Standards Institute, 2008), and for yeasts, document M27-A4 (Clinical and Laboratory Standards Institute, 2017) was referred to. The antifungal drugs included in AFST were nystatin (Nys), voriconazole (VCZ), itraconazole (ITR), fluconazole (FLZ), posaconazole (PSZ), amphotericin B (AmB), 5-fluorocytosine (5FC), and caspofungin (CAS) (all obtained from Sigma-Aldrich, St. Louis, MO, U.S.A). The quality control purposes involved the use of reference strains of *C. parapsilosis* (ATCC 22019) and *C. krusei* (ATCC 6258). The 530 nm wavelength was used to spectrophotometrically measure homogeneous conidial suspensions, with a percent transmission within the range of 75- 77%. The RPMI 1640 medium (GIBCO, UK) was used to adjust the final inoculum suspension to 0.5-2.5 × 10^3^ conidia/mL. To maintain a pH of 7.0, 0.165 M morpholino propane sulfonic acid (MOPS, Sigma-Aldrich, St. Louis, MO, USA) was used for buffering. Following the addition of 100 μL of the inoculum suspension, the microdilution plates were placed in an incubator at 35°C for 48 hours. Subsequently, the plates were visually examined following the guidelines outlined in the CLSI M27-A4 and M38-A2 documents.

### Determining hemolysin factor

The hemolysin assay for fungal strains was conducted using a previously validated protocol developed by Luo et al (Luo et al., 2001). To determine hemolysin production, SDA supplemented with 6% sheep blood and 3% glucose (pH= 5.6) was utilized. A yeast suspension (10^6^ cells/mL) was prepared in saline solution, and 10 μL was spot inoculated on sheep blood agar plates. The plates were then incubated at 37°C in 5% CO2 for 5 days. Afterward, we inspected the plates and calculated the hemolytic index (Hz value) by comparing the diameter of the colony with that of the translucent hemolysis zone (mm). The results were interpreted as high activity if ≤0.59; medium activity for 0.6–0.79; low activity for 0.8–0.99; and no activity for 1 (Canela et al., 2018). *C. albicans* (ATCC 14053) served as the positive control, whereas *Candida parapsilosis* (ATCC 22019) served as the negative control. Each isolate was tested in duplicate on three separate occasions.

### Determining proteinase activity

The proteinase activity of the isolates was evaluated following the method of Staib et al (Staib, 1969). A fungal suspension was prepared from overnight cultures, and 10 μL containing 1 × 10^6^ fungi cells/mL was used to inoculate the bovine serum albumin (BSA) agar plate. The BSA agar plate was composed of BSA solution 1%, dextrose 2%, KH2PO4 0.1%, MgSO4 0.05%, and agar 2%. After inoculation, the plates were incubated at 37°C for 72 h. Following this, 20% trichloroacetic acid was used to fix the plate for 15 minutes, and then it was stained with 1.25% amido black for 30 minutes. Afterward, we applied 15% acetic acid to remove the color from the setup before determining the zone (Pz) around the colonies. The hemolysin activity classification was used to represent the proteinase activity. As a positive control, *C. albicans* (ATCC 14053) was utilized, whereas *C. glabrata* (ATCC 90030) served as the negative control. Each isolate was subjected to the assay in duplicate on three separate occasions.

### Determining esterase activity

The esterase activity of the isolates was assessed using the Tween 80 opacity test, based on the method by Slifkin et al (Slifkin, 2000). A solution was prepared by dissolving 10 g peptone, 5 g NaCl, 0.1 g CaCl2, and 15 g agar in 1000 mL of distilled water, with the pH adjusted to 6.8, and then autoclaved. After cooling to 50°C, 5 mL of sterile Tween 80 was added to the media, which was then dispensed into 90 mm plates. Then, 10 microliters of fungal suspension (1 ×10^6^ cells/mL) were applied to each plate and incubated at 37°C for two days. The hemolysin activity classification was used to represent the esterase activity. *C. albicans* (ATCC 14053) served as the positive control. The assay was conducted twice on three separate occasions for each isolate.

### Statistical tests

The SPSS software (V.20) was used to conduct the data analysis. Standard Chi-squared and 95% Confidence intervals (CI) were utilized to assess the study. A statistically significant difference or correlation was indicated by a *P* value < 0.05. The virulence factors of the isolates were defined using simple frequencies. Furthermore, a comparison of continuous variables was carried out using the student’s t-test.

## Results

In total, 76 (36 males and 40 females) patients suffering from AD and 76 (41 males and 35 females) non-AD individuals were enrolled. Participants in the 2 studied groups were matched according to their age and gender (**Table 1**). The mean (SD) age of patients suffering from AD and non-AD individuals was 87.96 (7.91) and 85.18 (5.79) years, respectively (*p*=0.274) (**Table 1**).

**Table 1:** Demographic characteristics in the two groups studied.

Variable		AD patients (n = 76)	non-AD group (n = 76)	*p*-value ^a^
Age (years), mean (SD)		87.96 (7.91)	85.18 (5.79)	(*p*=0.274)
Sex, n (%)	Male Female	36 (47.3) 40 (52.7)	41 (53.9) 35 (46.1)	(*p*=0.615)
Result of fungal culture on SC, n (%)	Positive Negative	66 (86.9) 10 (13.1)	41 (53.9) 35 (46.1)	(*p*<0.001)

a Statistically significant difference between groups (*P*<0.05).

Analyzing the fungal species isolated from AD patients showed that *C. albicans* was the most frequently isolated species, accounting for 80% (n=51) of the cases, followed by *C. glabrata* at 9% (n=6). Similarly, in the non-AD, *C. albicans* dominated as the most frequently isolated species, representing 40% (n=28), followed by *C. glabrata* (n=8, 11%) and *C. parapsilosis* complex (n=8, 11%) (**Table 2**). The results highlighted a substantial difference between the AD patients and the non-AD individuals in terms of the prevalence of oral fungal composition (**Table 1**). The prevalence of oral fungal composition was found to be 1.6 times higher in AD patients in comparison with non-AD group (*P*<0.001). Among 76 studied AD patients 66 cases had positive results in fungal culture (66/76, 86.9%), whereas among 76 studied non-AD individuals 41 cases had positive results in fungal culture (41/76, 53.9%) (**Table 1**).

**Table 2:** The fungal species isolated from AD patients and non-AD individuals in the present study.

Studied group Fungal species	AD patients, n (%)	Non-AD group, n (%)	Total, n (%)
*C. albicans*	53 (80)	28 (40)	81 (59.6)
*C. glabrata*	6 (9)	8 (11)	14 (10.3)
*C. parapsilosis* complex	2 (3)	8 (11)	10 (7.4)
*P. kudriavzevii (C. krusei)*	2 (3)	5 (7.5)	7 (5.15)
*Cladosporium tenuissimum*	2 (3)	4 (5.5)	6 (4.4)
*Alternaria alternata*	1 (1)	3 (4.5)	4 (2.9)
*Aspergillus flavus*	0 (0)	3 (4.5)	3 (2.2)
*Aspergillus niger*	0 (0)	1 (1.5)	1 (0.74)
*Aspergillus fumogatus*	0 (0)	3 (4.5)	3 (2.2)
*Fusarium solani*	0 (0)	1 (1.5)	1 (0.74)
*Mucor circinelloides*	0 (0)	1 (1.5)	1 (0.74)
*Penicillium aurantiacum*	1 (1)	1 (1.5)	2 (1.5)
*Rhodotorula mucliginosa*	0 (0)	4 (5.5)	4 (2.9)
Total	66 (100)	70 (100)	136 (100)

The results of **Table 2** show that the diversity of the oral fungal composition in AD patients was less than non-AD individuals. From 66 AD patients with positive results in fungal culture, 66 fungal species were recovered, which means only one fungal species was isolated from the mouths of each patient suffering from AD (**Table 2**). While, from 41 non-AD patients with positive results in fungal culture, 70 fungal species were recovered, which means several fungal isolates were isolated from the mouths of each non-AD individual (**Table 2**).

**Table 3** shows the distribution of age, gender, body mass index (BMI), AD severity stages, and medications in AD patients with positive results in fungal culture and non-AD individuals with positive results in fungal culture.

**Table 3:** Demographic characteristics, underlying conditions, and medications in AD patients and non-AD individuals with positive results in fungal culture.

Variable		AD patients with positive results in fungal culture (n = 66)	non-AD individuals with positive results in fungal culture (n = 41)	*p*-value ^a^
Age (years), mean (SD)		85 (10.93)	82 (8.83)	(*p*=0.42)
Gender, n (%)	Male Female	30 (45.45) 36 (54.55)	19 (46.34) 22 (53.66)	(*p*=0.653)
BMI ^b^, mean (SD)	Normal (18.5–24.9 kg/m2) Overweight (25–29.9 kg/m2) Obese—Class 1 (30–34.9 kg/m2) Obese—Class 2 (35–39.9 kg/m2) Obese—Class 3 (≥40 kg/m2)	26.48 (4.29)	26.46 (4.03)	(*p*=0.801)
AD severity stages ^c^, n (%)	Group 1 Group 2 Group 3	21 (31.81) 26 (39.4) 19 (28.79)	-	(*p*=0.534)
Medications	History of receiving broad-spectrum antibiotics History of corticosteroid therapy	11 (16.66) 9 (13.64)	9(21.95) 6 (14.64)	(*p*=0.071)

a Statistically significant difference between groups (P<0.05).

b Body Mass Index.

c In the present study, the severity of AD was determined based on GDS, Reisberg Scale. In this classification, AD is divided into 7 stages based on the severity of the symptoms. With the knowledge that to be able to compare the cases statistically, we should have at least 10 cases in each stage of the disease, the stage 1 and 2 patients were categorized in Group 1, Stage 3 and 4 patients were categorized in Group 2, and Stage 5, 6, and 7 patients were categorized in group 3.

Statistical analysis of the results showed that there was no significant relationship between the prevalence of oral fungal composition and gender (*p*=0.694), BMI (*p*= 0.912), AD severity stage (*p*= 0.534), history of receiving broad-spectrum antibiotics (*p*= 0.215), and history of corticosteroid therapy (*p*=0.071) in AD patients and these underlying conditions did not affect the prevalence of oral fungal composition in these patients. Furthermore, the age of the patient had a significant effect on the prevalence of oral fungal composition in AD patients (*p*=0.004). Also, there was no statistical difference between the 2 studied groups (AD patients with positive results in fungal culture and non-AD individuals with positive results in fungal culture) based on the studied underlying conditions (for each *P*>0.05) (**Table 3**).

The activity of enzymes including esterase, hemolysin, and proteinase among the fungal species isolated from AD patients and non-AD individuals in the present study is shown in **Table 4**. From the 81 C*. albicans* isolates from AD patients and non-AD individuals, 29 isolates (35.8%) exhibited proteinase activity and among these, 19 isolates (65.51 %) were from AD patients, and 10 isolates (34.48 %) were from non-AD individuals. On the other hand, 52 C*. albicans* isolates (64.2%) did not exhibit proteinase activity.

**Table 4:** The activity of enzymes including esterase, hemolysin, and proteinase among the fungal species isolated from AD patients and non-AD individuals in the present study ^a^.

Virulence factor	Fungal isolates	AD patients (n = 66)		non-AD individuals (n = 41)		Total		Enzyme activity level		
		Positive	Negative	Positive	Negative	Positive	Negative	High	Medium	Low
Proteinase activity	*C. albicans* (n=81)	19	34	10	18	29	52	15	5	9
	*C. glabrata* (n=14)	5	1	3	5	8	6	5	0	3
	*C. parapsilosis* complex (n=10)	2	0	6	2	8	2	2	0	6
	*P. kudriavzevii* *(C. krusei)* (n=7)	2	0	2	3	4	3	2	0	2
	*Cladosporium tenuissimum* (n=6)	2	0	2	2	4	2	3	0	1
	*Alternaria alternata* (n=4)	1	0	1	2	2	2	0	0	2
	*Aspergillus flavus*(n=3)	0	0	0	3	0	3	0	0	0
	*Aspergillus niger(*n=1)	0	0	0	1	0	1	0	0	0
	*Aspergillus fumogatus*(n=3)	0	0	0	3	0	3	0	0	0
	*Fusarium solani (*n=1)	0	0	0	1	0	1	0	0	0
	*Mucor circinelloides* (n=1)	0	0	0	1	0	1	0	0	0
	*Penicillium aurantiacum* (n=2)	0	1	0	1	0	2	0	0	0
	*Rhodotorula mucliginosa* (n=4)	0	0	0	4	0	4	0	0	0
Mean EAI (mm)		0.89		0.92		*p*-value: 0.092				
Esterase activity	*C. albicans* (n=81)	8	45	6	22	14	67	2	2	11
	*C. glabrata* (n=14)	1	5	0	8	1	13	0	0	1
	*C. parapsilosis* complex (n=10)	2	0	1	7	3	7	2	0	1
	*P. kudriavzevii* *(C. krusei)* (n=7)	0	2	0	5	0	7	0	0	0
	*Cladosporium tenuissimum* (n=6)	0	2	0	4	0	6	0	0	0
	*Alternaria alternata* (n=4)	0	1	0	3	0	4	0	0	0
	*Aspergillus flavus* (n=3)	0	0	0	3	0	3	0	0	0
	*Aspergillus niger (*n=1)	0	0	0	1	0	1	0	0	0
	*Aspergillus fumogatus* (n=3)	0	0	0	3	0	3	0	0	0
	*Fusarium solani (*n=1)	0	0	0	1	0	1	0	0	0
	*Mucor circinelloides* (n=1)	0	0	0	1	0	1	0	0	0
	*Penicillium aurantiacum* (n=2)	0	1	0	1	0	2	0	0	0
	*Rhodotorula mucliginosa* (n=4)	0	0	0	4	0	4	0	0	0
Mean EAI (mm)		0.96		0.99		*p*-value: 0.133				
Hemolysin activity	*C. albicans* (n=81)	31	22	7	21	38	43	11	15	12
	*C. glabrata* (n=14)	5	1	0	8	5	9	3	2	0
	*C. parapsilosis* complex (n=10)	2	0	0	8	2	8	1	0	1
	*P. kudriavzevii* *(C. krusei)* (n=7)	2	0	0	5	2	5	1	0	1
	*Cladosporium tenuissimum* (n=6)	0	2	0	4	0	6	0	0	0
	*Alternaria alternata* (n=4)	0	1	0	3	0	4	0	0	0
	*Aspergillus. flavus*(n=3)	0	0	0	3	0	3	0	0	0
	*Aspergillus. niger(*n=1)	0	0	0	1	0	1	0	0	0
	*Aspergillus. fumogatus*(n=3)	0	0	0	3	0	3	0	0	0
	*Fusarium solani (*n=1)	0	0	0	1	0	1	0	0	0
	*Mucor circinelloides* (n=1)	0	0	0	1	0	1	0	0	0
	*Penicillium aurantiacum* (n=2)	0	1	0	1	0	2	0	0	0
	*Rhodotorula mucliginosa* (n=4)	0	0	0	4	0	4	0	0	0
Mean EAI (mm)		0.52		0.8		*p*-value: 0.04				

EAI, enzymatic activity index.

a Values are expressed as Number.

Among the 14 C*. glabrata* isolates from AD patients and non-AD individuals, 8 isolates (57.14 %) exhibited proteinase activity and among these, 5 isolates (38.47 %) were from AD patients, and 3 isolates (34.48 %) were from non-AD individuals. Also, 6 C*. glabrata* isolates (42.86 %) did not exhibit proteinase activity. Besides, among the 10 C*. pqarapsilosis* isolates from AD patients and non-AD individuals, 8 isolates (80 %) exhibited proteinase activity, 2 isolates (25 %) from AD patients, and 6 others (75 %) from non-AD individuals. Furthermore, 4 isolates of *P. kudriavzevii (C. krusei)* (57.14 %), 4 isolates of *Cladosporium tenuissimum* (66.67 %), and 2 isolates of *Alternaria alternata* (50 %) exhibited proteinase activity. It should be noted in the present study, the *Aspergillus flavus, A. niger, A. fumigatus, Fusarium solani, Mucor circinelloides, Penicillium aurantiacum*, and *Rhodotorula mucliginosa* isolates did not exhibit proteinase activity. The proteinase activity of all fungal species isolated in the present study is shown in **Table 4**. The mean activity of proteinase enzyme was measured to be 0.89 in the isolates from AD patients and 0.92 in the isolates from non-AD individuals, and there was not a statistically significant difference between the two groups (*p*= 0.092) (**Table 4**).

Among the 14 C*. albicans* isolates that had positive esterase activity, 8 isolates (57.14 %) were from AD patients, and 6 isolates (42.86 %) were from non-AD individuals. In the present study, from 14 C*. glabrata* isolates only 1 isolate (7.14 %) had esterase activity. From the 10 C*. parapsilosis* complex isolates from AD patients and non-AD individuals, 3 isolates (30%) exhibited esterase activity and among these, 2 isolates (66.67 %) were from AD patients, and 1 isolates (33.33 %) were from non-AD individuals. As shown in **Table 4** the other fungal isolates did not exhibit esterase activity. The mean Enzyme Activity Index (EAI) for esterase, was measured to be 0.96 in the isolates from AD patients and 0.99 in the isolates from non-AD individuals, and there was not a statistically significant difference between the two groups (*p*= 0.133) (**Table 4**).

The results of **Table 4** showed that the hemolysin enzyme activity in fungal species isolated from AD patients was statistically higher than that of the non-AD individuals (*P*= 0.04). The mean EAI for hemolysin in AD patients was 0.52, while in non-AD individuals, it was measured to be 0.8 (**Table 4**). Compared to non-AD individuals, the fungal species isolated from AD patients had considerably greater hemolysin activity (*p*= 0.04).

It should be noted in the current study, *C. parapsilosis complex* isolated from AD patients showed 100% proteinase, hemolysin, and esterase activities and had high levels of virulence factor activities in correlation with AD.

The results of antifungal susceptibility testing to nystatin (Nys), voriconazole (VCZ), itraconazole (ITR), fluconazole (FLZ), posaconazole (PSZ), amphotericin B (AmB), 5-fluorocytosine (5FC), and caspofungin (CAS) against 66 fungal strains obtained from AD patients are presented in **Table 5**. Antifungal susceptibility test showed that VCZ (MIC: 0.03-0.125 μg/mL), CAS (MIC range: 0.03-0.5 μg/mL), and ITZ (MIC range: 0.125-0.5 μg/mL) were the most active antifungals against *C. albicans* isolates and no resistance to these antifungal agents was observed.

**Table 5:** The results of *in vitro* susceptibility testing to nystatin (Nys), voriconazole (VCZ), itraconazole (ITR), fluconazole (FLZ), posaconazole (PSZ), amphotericin B (AmB), 5-fluorocytosine (5FC), and caspofungin (CAS) against 66 fungal strains obtained from AD patients.

Strains	Antifungals	MIC range	CLSI M27-A4 and CLSI M38-A2 Breakpoints (n)		
			R	SDD/I	S
*C. albicans* (n=53)	Nys	1-4	2	-	51
	VCZ	0.03-0.125	-	-	53
	ITZ	0.125-0.5	-	1	52
	FLZ	0.25-64	15	4	34
	PSZ	0.03-0.125	1	-	52
	AmB	0.06-2	25	-	28
	5FC	0.5-2	2		51
	CAS	0.03-0.5	-	2	51
*C. glabrata* (n=6)	Nys	1-2	-	-	6
	VCZ	1-2	-	1	5
	ITZ	0.125-1	1	1	4
	FLZ	0. 125-4	-	1	5
	PSZ	0.03-0.125	3	-	3
	AmB	0.06-1	1	-	5
	5FC	≤0.03-0.5	-	-	6
	CAS	≤0.03-0.125	-	-	6
*C. parapsilosis* complex (n=2)	Nys	1-2	-	-	2
	VCZ	0.125	-	-	2
	ITZ	0.125	-	-	2
	FLZ	0.25-0.5	-	-	2
	PSZ	0.06	-	-	2
	AmB	0.25	-	-	2
	5FC	0.125	-	-	2
	CAS	0.25	-	-	2
*P. kudriavzevii* *(C. krusei)* (n=2)	Nys	1	-	-	2
	VCZ	≤0.03-0.03	-	-	2
	ITZ	0.125	-	-	2
	FLZ	0.25	-	-	2
	PSZ	0.03	-	-	2
	AmB	2	2	-	-
	5FC	16	2	-	-
	CAS	0.25	-	-	2
*Cladosporium tenuissimum* (n=2)	Nys	>16	-	-	-
	VCZ	16	-	-	-
	ITZ	0.25–0.5	-	-	-
	FLZ	0.5–8	-	-	-
	PSZ	0.25–0.5	-	-	-
	AmB	0.5–1	-	-	-
	5FC	1–16	-	-	-
	CAS	4–8	-	-	-
*Alternaria alternata* (n=1)	Nys	0.5	-	-	-
	VCZ	0.06	-	-	-
	ITZ	0.06	-	-	-
	FLZ	4	-	-	-
	PSZ	0.25	-	-	-
	AmB	0.03	-	-	-
	5FC	2	-	-	-
	CAS	0.06	-	-	-
*Penicillium aurantiacum* (n=1)	Nys	>16	-	-	-
	VCZ	0.25	-	-	-
	ITZ	0.06	-	-	-
	FLZ	0. 5	-	-	-
	PSZ	0.5	-	-	-
	AmB	0.06	-	-	-
	5FC	8	-	-	-
	CAS	0.03	-	-	-

MIC, Minimum inhibitory concentration; R, resistant; SDD, susceptible-dependent-dose; I, intermediate; S, susceptible; Nys, nystatin; AMB, Amphotericin B; FLZ, Fluconazole; ITZ, itraconazole; PSZ, posaconazole; 5FC, 5-fluorocytosine; VOR, voriconazole; CAS, caspofungL. No clinical breakpoints (CBP) or epidemiologic cut-of values (ECV) had been identified for nystatin by CLSI. As an alternative, ECV for amphotericin B has been used as a cut-off value to separate susceptible and resistant isolates (Pfaller and Diekema, 2012).

Also, CAS (MIC: ≤0.03-0.125 μg/mL), 5FC (MIC range: 0.03-0.5 μg/mL), and Nys (MIC range: 1-2 μg/mL) were the most active antifungals against *C. glabrata* isolates and no resistance to these antifungal agents was observed.

*C. parapsilosis* complex isolates (n=2) were susceptible to all antifungal agents. Although, *P. kudriavzevii (C. krusei)* isolates (n=2) were 5FC-resistant (MIC: 16 μg/mL) and AmB-resistant (MIC: 2 μg/mL).

The results showed that ITZ (MIC range: 0.25-0.5 μg/mL) and PSZ (MIC range: 0.25-0.5 μg/mL) had the greatest and Nys (MIC range: >16 μg/mL had the lowest antifungal activity against *Cladosporium tenuissimum* isolates (n=2). Also, AmB (MIC: 0.03 μg/mL) had the greatest, and FLZ (MIC: 4 μg/Ml) had the lowest antifungal activity against *Alternaria alternate* (n=1). Furthermore, CAS (MIC: 0.03 μg/mL) had the greatest, and Nys (MIC: >16 μg/Ml) had the lowest antifungal activity against *Penicillium aurantiacum* (n=1).

## Discussion

The oral cavity is home to the second largest and most diverse microbiota after the intestine, with more than 700 species of bacteria, viruses, and fungi (Ramezanalipour et al., 2024). Oral-associated microbes have also been found in many distant organs, including the small intestine, lungs, heart, placenta, and brain (Sedghi et al., 2021). Many associations have been noted between oral microorganisms, including those associated with periodontal disease, and other widespread chronic illnesses such as cardiovascular disease, hypertension, and high blood pressure (Del Pinto et al., 2020). Some patients with AD have experienced a reduction in clinical symptoms as a result of antifungal treatment (Ala et al., 2004; Hoffmann et al., 2009). Furthermore, the cytokine and inflammatory molecule profiles identified in AD patients bore similarities to those caused by fungal infections (Behrens et al., 2019; Ganguly et al., 2021).

Early studies indicate that microorganisms found in the oral cavity have the potential to impact immune reactions and the development of diseases beyond the oral cavity (Li et al., 2022). On the other hand, the human mycobiome, like the microbiome, is characterized by its dynamic nature, exhibiting significant variability across different stages of life, both at the intra- and inter-individual levels (Revel-Muroz et al., 2023). To the best of our knowledge, the present investigation is the first study conducted on investigating the changes in the frequency of oral fungal composition, testing the antifungal susceptibility, and assessing the pathogenesis profiles of isolated species in patients suffering from AD compared to the non-AD individuals.

The results showed that compared to the non-AD group, the prevalence of oral fungal composition in AD group was 1.6 times higher. Lack of proper oral and dental hygiene, increased risk of periodontal disease, bleeding gums, xerostomia (dry mouth), decreased saliva secretion, and gum infections can be the reasons for the higher frequency of oral fungal composition in AD patients compared to the non-AD group.

*C. albicans* was the most common fungal species isolated from oral swab samples of AD group followed by *C. glabrata*, *C. parapsilosis*, *P. kudriavzevii*, *Cladosporium tenuissimum*, *Alternaria alternate*, and *Penicillium aurantiacum*. Studies have shown that induction of IL-17 is instructed by dendritic cells (in particular by the Langerin+ subset of dendritic cells). These cells have the unique property in the *C. albicans*-infected oral mucosa to co-produce the three major IL-17-instructing cytokines IL-23, IL-1, and IL-6 (Buccellato et al., 2021; Lionakis et al., 2023). These cytokines play an important role in inflammation. Furthermore, results of a study conducted by Ghannoum et al., found a total of 85 fungal genera in the oral mycobiome (Ghannoum et al., 2010). The most frequent genera included *Candida*, *Cladosporium*, *Aureobasidium*, *Saccharomycetales*, *Aspergillus*, *Fusarium*, and *Cryptococcus*, of which four of these predominant genera are known human pathogens (Ghannoum et al., 2010). Results of a study conducted by Phuna et al., showed that *C. albicans* is frequently detected in the brain of AD patients (Phuna and Madhavan, 2022). Also, the Aβ protein has shown to display antimicrobial characteristics when exposed to this fungus (Phuna and Madhavan, 2023). At this stage, the DNA of fungi remains undetectable yet active. This can act as the prolonged pathogenic stimulus that over-triggers the expression of Aβ-related genes, which subsequently lead to overproduction and deposition of Aβ plaque and neuroinflammation. Also, several fungal macromolecules including fungal proteins and polysaccharides ((1,3)-β-glucan) have been detected in peripheral blood serum from AD-patients, and fungal proteins were found in brain tissue (Yashkin et al., 2022).

The results of the present study showed that the composition and diversity of the oral fungal microbiome in AD patients was less than non-AD individuals suggesting an overgrowth of specific fungal species in AD patients. This dysbiosis (an imbalance in the oral microbiome, where certain microorganisms become overabundant) leading to a heightened inflammatory response within the mouth, which can potentially contribute to the progression of the disease through systemic inflammation due to the interaction between oral residents and the immune system (Radaic and Kapila, 2021).

*C. albicans*, *C. glabrata*, *C. parapsilosis* complex, *P. kudriavzevii* (*C. krusei*), *Cladosporium tenuissimum*, *Alternaria alternata*, and *Penicillium aurantiacum* were the oral fungal microbiome isolated from AD patients. Whereas, in addition to the above-mentioned fungal species, *Aspergillus flavus*, *Aspergillus niger*, *Aspergillus fumigatus*, *Fusarium solani*, *Mucor circinelloides*, and *Rhodotorula mucliginosa* were also isolated from non-AD individuals. Da et al. demonstrated that patients with AD have lower oral microbiome diversities compared to those who are cognitively healthy (Da et al., 2023).

In the present study, the activity of enzymes including esterase, hemolysin, and proteinase among the fungal species isolated from AD patients and non-AD individuals was measured and showed some fungal microbiome had high levels of virulence factor activity compare to others belonging to the same fungal species. The expression of pathogenesis factors in fungi is influenced by environmental factors like temperature, nutrient availability, host immune response, pH levels, and the presence of specific signaling molecules. Also, the age of the fungal cell can be another influential factor (Canela et al., 2018). Besides, the results showed that among the studied virulence factors, the average hemolytic activity of fungal species isolated from the oral samples of AD patients was significantly higher than non-AD group. Hemolysins are enzymes capable of lysing red blood cells through the destruction of the cell membrane, leading to the release of iron. The rise in iron levels and its release into the environment of the fungal isolates leads to an increase in fungal growth, consequently facilitating the invasion of the fungi into the oral tissue (Kosmachevskaya et al., 2021; Vallelian et al., 2022).

On the other hand, the rise of antifungal drug resistance among fungal opportunists poses a growing challenge in modern medicine. The implementation of potent antifungal medications could potentially help alleviate this issue (Vitiello et al., 2023).. In this study, we tested the *in vitro* activities of 8 antifungal agents (nystatin, voriconazole, itraconazole, fluconazole, posaconazole, amphotericin B, 5-fluorocytosine, and caspofungin) against the against 66 fungal strains obtained from AD patients. Although, nystatin (a broad spectrum polyene antifungal drug) is the drug of choice for the treatment of superficial *Candida* infections of the mucous membranes of mouth, in the present study 1.69 % (2/118) of the *Candida* isolates were resistant to this antifungal drug (Sousa et al., 2023). Also, a low MIC (0.5 μg/mL ) was obtained for nystatin against the saprophytic fungi, *Alternaria alternate*. Nystatin works by attaching to ergosterol in the fungal cell membrane, leading to a disruption in the membrane's permeability and exerting its antifungal effect. Mammalian cells are not affected by the drug due to the absence of ergosterol in their cell membrane (Rai et al., 2022; Sousa et al., 2023). Nystatin exhibits fungistatic or fungicidal effects against a range of pathogenic and nonpathogenic yeast and fungal strains. This polyene antibiotic shares structural similarities with amphotericin B, but its limited oral bioavailability restricts its use to topical applications (Rai et al., 2022; Sousa et al., 2023). The current research illustrated that nystatin prophylaxis could be deemed as a successful medication in preventing fungal colonization and potentially reducing the symptoms or advancement of AD due to its safety, tolerability, affordability, and efficacy. However, since this was an *in-vitro* study, a clinical trial would be more beneficial in aiding clinicians to determine the optimal prophylaxis protocols.

The study did not investigate aspects related to Candidalysin, biofilm formation and some other significant factors contributing to the pathogenicity of fungal species. The presence of these factors might have implications for oral fungal composition dynamics in AD patients, and not addressing these aspects could be considered a limitation. Also, the present study did not show any type of correlation between fungal colonization of oral mucosal surfaces and neurodegenerative disorders. Furthermore, it did not show any correlation between disease states and the fungal virulence factors. Therefore, it is suggested to carry out studies in this field with a larger sample size in the future.

## Conclusions

The current study has demonstrated that the prevalence of oral fungal composition in AD–patients was 1.6 times higher than non-AD individuals. *Candida albicans* was the most common fungal species isolated from oral swab samples of AD group and the diversity of the oral fungal composition in AD-patients were lower than non- AD individuals. Among the 3 investigated virulence factors (esterase, hemolysin, and proteinase), a statistically significant difference was shown in terms of hemolysin activity level between the two studied groups. The present showed that nystatin prophylaxis might be considered an effective drug in the prevention of fungal colonization and might lower the clinical manifestations or progression of AD.

## Data Availability

The datasets presented in this study can be found in online repositories. The names of the repository/repositories and accession number(s) can be found in the article/supplementary material.
